# Optimal slice thickness for cone-beam CT with on-board imager

**DOI:** 10.2349/biij.6.3.e31

**Published:** 2010-07-01

**Authors:** KYT Seet, A Barghi, S Yartsev, J Van Dyk

**Affiliations:** London Regional Cancer Program, London Health Sciences Centre, London, Ontario, Canada

**Keywords:** Cone beam CT, slice thickness, automatic registration, contrast-to-noise ratio, on-board imager

## Abstract

**Purpose:**

To find the optimal slice thickness (*Δτ*) setting for patient registration with kilovoltage cone-beam CT (kVCBCT) on the Varian On Board Imager (OBI) system by investigating the relationship of slice thickness to automatic registration accuracy and contrast-to-noise ratio.

**Materials and method:**

Automatic registration was performed on kVCBCT studies of the head and pelvis of a RANDO anthropomorphic phantom. Images were reconstructed with 1.0 ≤ *Δτ* (mm) ≤ 5.0 at 1.0 mm increments. The phantoms were offset by a known amount, and the suggested shifts were compared to the known shifts by calculating the residual error. A uniform cylindrical phantom with cylindrical inserts of various known CT numbers was scanned with kVCBCT at 1.0 ≤ *Δτ* (mm) ≤ 5.0 at increments of 0.5 mm. The contrast-to-noise ratios for the inserts were measured at each *Δτ*.

**Results:**

For the planning CT slice thickness used in this study, there was no significant difference in residual error below a threshold equal to the planning CT slice thickness. For *Δτ > *3.0 mm, residual error increased for both the head and pelvis phantom studies. The contrast-to-noise ratio is proportional to slice thickness until *Δτ* = 2.5 mm. Beyond this point, the contrast-to-noise ratio was not affected by *Δτ*.

**Conclusion:**

Automatic registration accuracy is greatest when 1.0 ≤ *Δτ* (mm) ≤ 3.0 is used. Contrast-to-noise ratio is optimal for the 2.5 ≤ *Δτ* (mm) ≤ 5.0 range. Therefore 2.5 ≤ *Δτ* (mm) ≤ 3.0 is recommended for kVCBCT patient registration where the planning CT is 3.0 mm.

## INTRODUCTION

Image-guided radiation therapy (IGRT) is carried out to improve treatment accuracy in patients. The Varian on-board imaging (OBI) system is an IGRT modality that permits kilovoltage cone-beam CT on the treatment machine. kVCBCT obtains 3D volumetric studies of patients in the treatment room immediately before radiation delivery. The main advantage of cone-beam CT is the minimisation of patient movement between imaging and treatment, such that dose gradients conform to planning target volumes. These images, although prone to artefacts such as streaking and cupping effects, can still be used to ensure accurate and consistent patient positioning prior to treatment. kVCBCT is an ideal tool for fast patient registration, only requiring one 360° rotation of the gantry (t = 60 s) for acquisition. Both the kVCBCT and treatment beam (MV) are commissioned to share the same isocentre. The OBI software performs an automatic registration of cone-beam CT studies to match planning CT and the patient can be shifted accordingly with reference to the isocentre. Previous investigations on the usage of megavoltage CT (MVCT) studies for daily set-up on helical tomography have found the automatic registration process to be an adequate procedure [1, 2]. In volumetric CT studies, the axis of gantry rotation is referred to as the longitudinal axis. The resolution along this axis is referred to as the ‘slice thickness’ (*Δτ*) of the CT study. In planning CT, *Δτ* has a significant effect on how much radiation is delivered to the patient during the imaging session [3]. Smaller *Δτ* can therefore only be achieved at the price of increased imaging dose in planning CT. To increase resolution, interpolation of acquired planning CT data is possible, but it causes aliasing artefacts [4]. Therefore, the longitudinal direction has the lowest resolution in planning CT studies. Unlike planning CT, kVCBCT always acquires all its information in a single gantry rotation regardless of slice thickness. Selecting a different kVCBCT slice thickness only affects how the information is partitioned from the flat-panel detector during reconstruction, and has no influence on the method of image acquisition or amount of radiation delivered by the x-ray generator. For reconstruction of the kVCBCT image using the Varian OBI system, *Δτ* ranging from 1 to 5 mm can be selected at increments of 0.5 mm. This study focuses on automatic registrations using planning CT at 3 mm slice spacing, to evaluate the consequences, if any, of increasing *Δτ* greater than that of the reference set, as well as the potential advantages of using *Δτ *smaller than the resolution of the reference set. A motivation for this investigation comes from megavoltage CT investigations on automatic registration accuracy, which have found that decreasing slice thickness past a certain value offers no advantage since registration accuracy becomes very similar [1]. Sykes *et al.* investigated automatic registration of kVCBCT with planning CT of various beam energy and slice thickness settings for the Synergy system (Elekta, UK) [5]. At the London Regional Cancer Program (LRCP), a standard 3 mm slice thickness is used clinically for planning CT imaging. This study aims to explore the optimal *Δτ* value for kVCBCT image reconstruction to achieve optimal image registration with 3 mm planning CT studies and image quality (defined by contrast-to-noise ratio).

Cone beam images generally produce higher noise (standard deviation in HU) in kVCBCT studies compared to planning CT studies when imaged with a similar set of scan parameters. Studies have shown that noise levels seen in kVCBCT images do not cause a significant loss in automated registration accuracy [6]. However, for the manual registrations, *Δτ* choice is important because the apparent “visibility” can be improved with an optimal setting, making image noise an important factor to investigate when considering *Δτ* optimisation. Resolution and noise trade-offs have been observed in previous investigations with fan-beam CT [7, 8]. Lowering the resolution by increasing *Δτ* may reduce noise in kVCBCT images in cases where longitudinal variation in CT numbers is minimal. If automatic registration accuracy is not affected by slice thickness, as is the case in MVCT investigations, reducing noise with kVCBCT would be possible with no loss of automatic registration accuracy.

## MATERIALS AND METHODS

### Registration accuracy vs. slice thickness

The OBI system consists of a kV x-ray source and flat panel detector mounted orthogonal to the treatment beam axis. For this study, registration accuracy was investigated by performing automatic registrations to the head and pelvis of a RANDO anthropomorphic male phantom, manufactured by The Phantom Laboratory (Salem, NY). The RANDO phantom is a 73.5 kg male human mould of synthetic soft tissue-equivalent material with real human skeleton. Planning CT studies of the phantom were acquired with a Philips PQ5000 CT simulator with 120 kV, 85 mA, 3 mm slice spacing, 25 cm FOV and 512 × 512 slice resolution (typical clinical setting) and transferred to the Eclipse planning system via the RT DICOM protocol. This investigation focused on 3 mm slice spacing for planning CT as the reference set following the authors’ institution practice, which is the median value of *Δτ* for OBI reconstruction. The head and pelvis of the phantom were aligned to the isocentre of the OBI system using the room’s lasers and metal ball bearings that were attached to the phantom during planning CT. The phantoms were then offset by a known amount in the lateral (Δx), superior-inferior (Δy), and anterior-posterior (Δz) directions. In this offset position, a kVCBCT study was acquired at 125 kVp, 80 mA, and 25 ms. Fields of view of 25 × 25 cm^2^ and 50 × 50 cm^2^ were used for the head and pelvis of the phantom, respectively. Slices were reconstructed at 512 × 512 pixels. A bowtie filter, which is an aluminium attachment that covers the x-ray source, was used to reduce artefacts, as recommended by the manufacturer.

With the Varian OBI software, kVCBCT and planning CT studies can be viewed overlapping one another. The user has the option of manual or automatic registration. The automatic registration algorithm uses a similarity measure cost function to find the global maximum to match pixel intensities [9]. Correctional shifts are displayed with 1 mm accuracy, which is the same as the resolution of the couch position. For five values of slice thickness *Δτ* ranging from 1 to 5 mm, automatic matches were performed and the suggested shifts were recorded and compared to the known shifts for both the head and pelvis of the phantom. Residual error was calculated using:

(1) $$R=\sqrt{{\left(x_{t}-\Delta x\right)}^{2}+{\left(y_{t}-\Delta y\right)}^{2}+{\left(z_{t}-\Delta z\right)}^{2}}$$

where R is the residual error, x*_t_*, y*_t_*, and z*_t _*are the suggested shifts given by the automatic match, and Δx, Δy, and Δz are the known shifts. To account for set-up errors, phantoms were returned to an offset position and the automatic registration process repeated four times. The residual error was then averaged.

### Image noise vs. slice thickness

A CatPhan phantom, a cylindrical phantom of radius 10 cm containing cylindrical inserts of radius 0.6 cm and of varying densities, manufactured by The Phantom Laboratory (Salem NY), was scanned with kVCBCT using a bowtie filter at 125 kVp, 80 mA, and 25 ms, and reconstructed at 5-slice thickness in the range of 1 ≤ *Δτ* (mm) ≤ 5. Inserts with known CT numbers (−1000, −200, −100, 340, and 990 HU) were compared to the CatPhan’s uniform background of 100 HU. Using ImageJ [10], an image-processing program developed by The National Institutes of Health, the CT numbers of the inserts (on the central slice) and their surrounding background were measured. For each insert, the average CT number and standard deviation of a circular area of radius 0.6 cm was determined. A ring of outer radius 1.0 cm and inner radius 0.8 cm was used around each insert to characterise the background. Contrast-to-noise ratios were calculated using relation:

(2) $$CNR=2\frac{HU_{ins}-HU_{surr}}{\sigma_{ins}+\sigma_{surr}}$$

where CNR is the contrast-to-noise ratio, HU_ins_ is the average CT number of the insert, HU_surr_ is the average CT number of the surroundings, σ_ins_ is the standard deviation of the CT number of the insert, and σ_surr_ is the standard deviation of the CT number of the surroundings.

## RESULTS AND DISCUSSION

**Figure 1:**
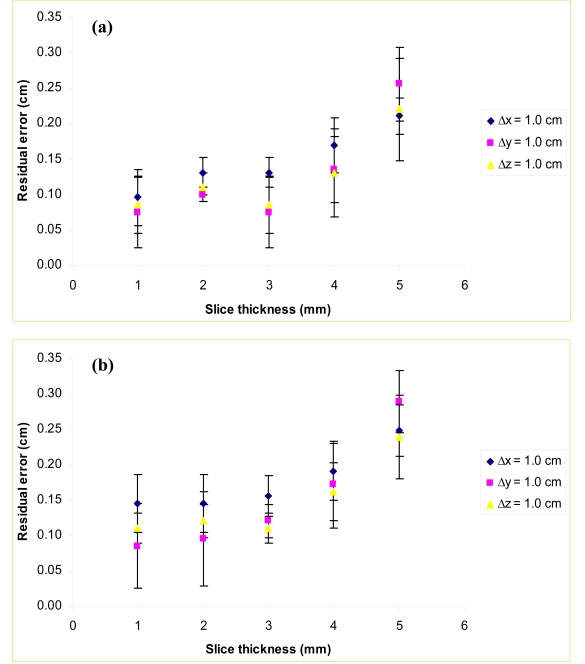
Residual errors vs. slice thickness of the RANDO (a) head phantom and (b) pelvis phantom registered at initial translations in the x, y, and z directions.

The residual errors of the automatic registrations calculated using eq. (1) from the measurements on the head and pelvis of the RANDO phantom for varying *Δτ* are presented in Figures 1(a) and 1(b), respectively. For *Δτ* ≤ 3 mm, there is no significant difference in residual error. For *Δτ* > 3 mm, residual error increases. The increase in residual error is greatest when the initial translation is in the longitudinal direction. This is the direction in which changing *Δτ* changes the resolution. *Δτ* = 3.0 mm is the limit at which *Δτ* can be increased without affecting the accuracy of the automatic registration. This is because the planning CT was taken at a slice spacing of 3.0 mm, which suggests that there is no advantage for automatic registration to use a resolution lower than that of the reference set. This may be a consequence of the registration algorithm, which is limited by the finite axial resolution of the planning CT. Reconstruction occurs before automatic registration, meaning that CBCT data is reconstructed into finite axial slices before the automatic registration algorithm is used, but the algorithm can interpolate the CBCT data to achieve maximum overlap. However, the planning CT slices are fixed during this process, so registration accuracy will not improve past a certain point by changing the resolution of the CBCT data alone. These results show that the automatic registration procedure is optimal when *Δτ* ≤ 3 mm, with no advantage of one slice thickness over another within this range.

**Figure 2:**
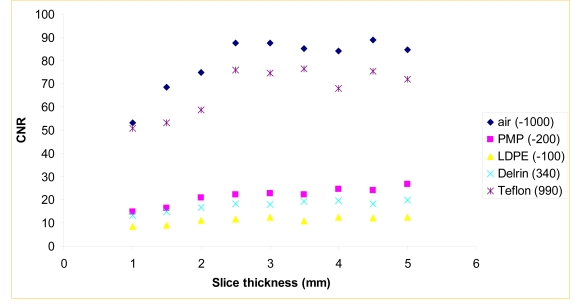
Contrast-to-noise ratio vs. slice thickness for inserts of air, polymethylpentene, low-density polyethylene, Delrin, and Teflon within a CatPhan phantom.

The contrast-to-noise ratio was determined by the measurement of insert of various CT numbers. Figure 2 shows the dependence of contrast-to-noise ratio on slice thickness for five different inserts within a CatPhan phantom. CNR increases steadily within the range 1.0 ≤ *Δτ* (mm) ≤ 2.5, then saturates for greater values. This is due to the low resolution of the kVCBCT flat panel detector with rectangular pixels of 0.2 × 0.4 mm^2^. A reduction of pixels in one direction also affects the smoothing of noise.

**Figure 3:**
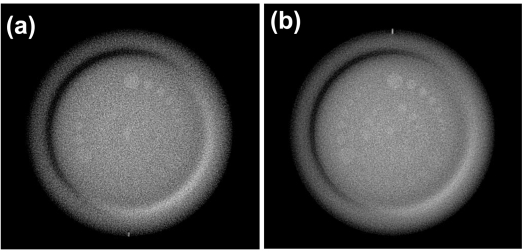
kVCBCT slices of CatPhan phantom with low contrast inserts reconstructed at (a) 1.0 and (b) 2.5 mm slice thickness. Window and level are the same for both images.

Figure 3 shows kVCBCT slices of the CatPhan phantom with low contrast inserts reconstructed at 1.0 mm and 2.5 mm slice thicknesses. This demonstrates the significance of the CNR decrease seen at low *Δτ* with kVCBCT, where noise levels in the 1.0 mm reconstructed slice are high enough that the visibility of some smaller inserts becomes less. Reconstruction of the CatPhan at *Δτ* ≥ 2.5 mm therefore improved visibility of low contrast structures.

Inserts were also located within the central portion of the phantom where the background HU remained constant (within ±5 HU). Depending on the field of view and object size, reconstructed images can exhibit streaking and cupping artefacts, resulting in CT number inaccuracy [11]. In this study, measurements were not affected by these artefacts due to imaging parameters and phantom size.

In this study, pCT images of the head and pelvis of the RANDO phantom were captured at 3.0 mm slice thickness as this was the clinical standard for slice thickness. Decreasing this slice thickness is likely to produce registrations of greater accuracy. However, results in this study demonstrate the best outcome for least accuracy in slice thickness. One of the new features of the recent OBI version1.4 is the automatic registration with 2.5 mm slice. The selection of planning CT slice thickness should therefore be considered if clinics decide to use automated registration functions.

## CONCLUSION

The head and pelvis of a RANDO anthropomorphic phantom were used to find an optimal 1.0 ≤ *Δτ* (mm) ≤ 3.0 for the automatic registration procedure when using 3.0 mm slice spacing with planning CT. This study shows that using *Δτ* smaller than the reference set offers no advantage. Image noise is also a function of slice thickness. An optimal slice thickness range for reducing image noise of 2.5 ≤ *Δτ* (mm) ≤ 5.0 was also found using a CatPhan phantom. The overlap between these two ranges is 2.5 ≤ *Δτ *(mm) ≤ 3.0, providing optimal automatic registration accuracy and visibility.
